# Age- and sex-specific reference intervals for blood urea nitrogen in Chinese general population

**DOI:** 10.1038/s41598-021-89565-x

**Published:** 2021-05-12

**Authors:** Qingquan Liu, Yiru Wang, Zhi Chen, Xiaolin Guo, Yongman Lv

**Affiliations:** 1 Department of Nephrology, Tongji Hospital, Tongji Medical College, Huazhong University of Science and Technology, Wuhan, 430030 People’s Republic of China; 2 Department of Geriatrics, Tongji Hospital, Tongji Medical College, Huazhong University of Science and Technology, Wuhan, 430030 People’s Republic of China; 3 Department of Urology, Tongji Hospital, Tongji Medical College, Huazhong University of Science and Technology, No.1095 Jiefang Avenue, Wuhan, 430030 Hubei People’s Republic of China; 4 Department of Health Management Centre, Tongji Hospital, Tongji Medical College, Huazhong University of Science and Technology, Wuhan, 430030 People’s Republic of China

**Keywords:** Biomarkers, Medical research, Nephrology, Urology

## Abstract

Blood urea nitrogen (BUN) is a nitrogenous end product of protein metabolism. This study aims to explore the age- and sex-specific distribution of BUN among healthy Chinese adults. A total of 24,006 BUN values from healthy adults (14,148 males and 9858 females) were included in the cross-sectional study. Males had a higher median BUN value compared to females (4.6 mmol/L *vs.* 4.1 mmol/L). BUN values showed a positive correlation with body mass index (BMI), cholesterol, and blood sugar (P < 0.0001). However, eGFR showed a negative correlation with the BUN reference value (P < 0.0001) in both sexes. Multiple linear regression analysis confirmed that the positive associations of BUN levels and age were statistically significant after adjusting confounding factors (*P* < 0.001). Thus, the serum BUN values increased by 0.21 mmol/L for males and 0.282 mmol/L for females per 10 years. The BUN values corresponding to the 1st, 2.5th, 50th, 97.5th, and 99th percentiles for any specific age in both sex were also calculated. These results indicate that the serum BUN reference value is significantly affected by age and gender, and thus, its interpretation is age- and sex-dependent.

## Introduction

Blood urea nitrogen (BUN) is a nitrogenous end product of protein metabolism and also produced in the liver by the breakdown of dietary proteins. The blood BUN level is mainly dependent on its rate of glomerular filtration and tubular reabsorption. Therefore, BUN plays a vital role in diagnosing and evaluating renal function^1^. Besides, Klein et al. found that BUN predicts survival in patients admitted with heart failure (HF) and not creatinine^2^. They suggested that BUN plays an essential role in predicting cardiovascular events caused by acute heart failure and is a prognostic or a neurohormonal activation biomarker in heart failure^3,4^.

Several studies reported that environmental and other factors, such as geographical change, climate change, gender, age, dietary structure, physical functioning affect BUN values^5^. With an increase in age, there is a sex difference in protein turnover. Some studies suggested that elderly females showed a higher protein synthesis rate compared to males, although females have less muscle mass^6,7^. These findings indicate that the level of BUN in serum may change dynamically with age. However, the value of serum BUN between males and females differ, irrespective of the same age group. Few studies have been performed to establish reference intervals for serum BUN, taking into account the difference between gender and age^8^.

At present, the clinical reference range value of BUN is non-standard and not uniform, both nationally and abroad. Most clinical laboratories establish the reference intervals from medical literature or the diagnostic test manufacturer^9,10^. To accurately diagnose the patient's condition and avoid subjective factors, it is necessary to evaluate accurately whether the BUN value is within the normal range. Thus, establishing age- and gender-specific reference intervals for BUN for the local population is critical and of utmost importance.

## Materials and methods

The study was performed at the Department of Health Management Center and approved by the Ethics Committee of Tongji Hospital, Huazhong University of Science and Technology. The included study subjects aged between 14 and 85 years and were considered healthy after a physical examination. For participants less than 18 years old, their informed consent was obtained from their parents or legal guardians. The exclusion criteria included < 60 mL/min/1.73 m^2^ estimated glomerular filtration rate (eGFR), diabetes mellitus, severe (acutely or chronically) infections, hyperthyroidism, liver cirrhosis, hepatic insufficiency, heart failure, chronic severe anemia, tumor, vegetarian, and pregnant woman. BUN values of a total of 22,006 subjects were collected in the cross-sectional study.

### Statistical analysis

Statistical analysis was performed using SPSS 17.0 (SPSS, Chicago, Illinois, USA) and R V.3.6.2 (R Foundation for Statistical Computing, Vienna, Austria). Data were expressed as mean ± SD for normally distributed variables and as median with IQR for non-normally distributed data. Categorical data were expressed as numbers and percentages. The data were analyzed by Pearson’s correlation to determine the correlation between serum BUN reference value and other variables. Multiple linear regression analysis was assessed to evaluate the relationship between BUN and the clinical variables. A two-tailed p-value < 0.05 was considered to be statistically significant. The 1st, 2.5th, 97.5th, and 99th percentiles of BUN values were estimated and provided 90% reference intervals. Confidence intervals (90%) for these percentiles across the age used bootstrap. The variance inflation factors (VIF) were calculated to determine multicollinearity among the variables. A (VIF) > 10 indicated collinearity among the variables^11^.

### Ethical approval and informed consent

The study was approved by the Ethics Committee of Tongji Hospital, Tongji Medical College, Huazhong University of Science and Technology (The Institutional Review Board Approval Number: TJ-C20160115). The study conforms to the principles outlined in the Declaration of Helsinki and written informed consent was obtained from all participants.

## Results

### Population characteristics

**Figure 1:**
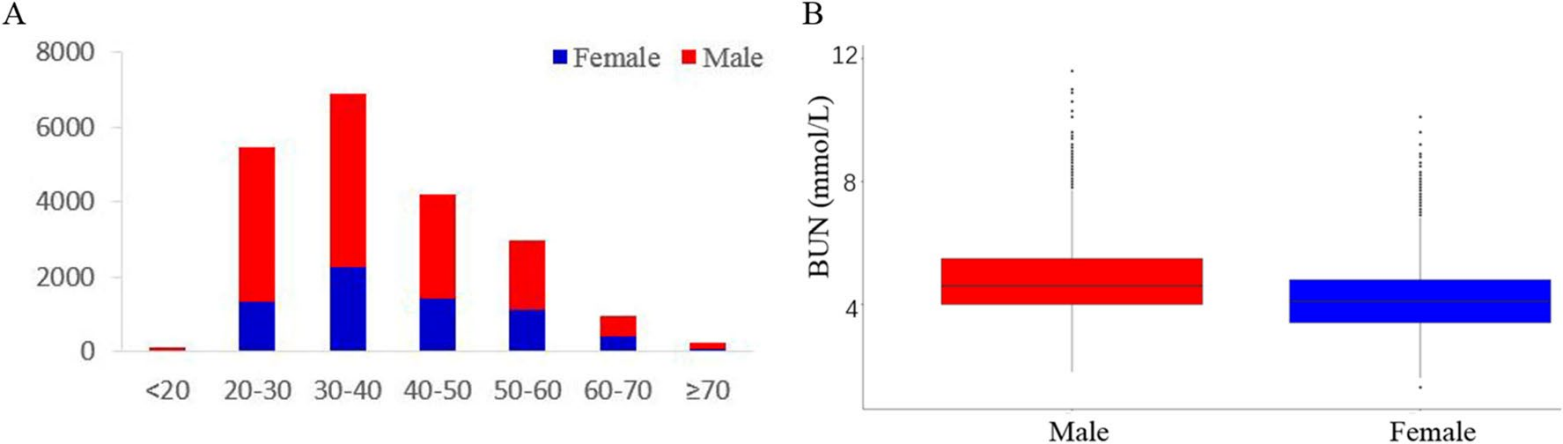
(**A**) Histogram for age and gender distribution of the BUN reference in males and females population. (**B**) Baseline BUN in males and females population. The box represents IQRs, the horizontal line in each box represents the median and the whiskers show the 10–90 percentile range.

The population under the study consisted of 14,148 males and 9858 females. The baseline characteristics of the male and female groups are described in Table 1. Males have higher BMI, blood pressure, total cholesterol, uric acid, as well as BUN, and lower estimated glomerular filtration rate (eGFR), compared to females. There was no significant difference in age and glucose between the two groups. Figure 1A illustrates a histogram for the age and gender distribution of the population. The median BUN values in males and females from the general population were 4.6 mmol/L (IQR: 4.0–5.5 mmol/L) and 4.1 mmol/L (IQR: 3.4–4.8 mmol/L), respectively (Fig. 1B).

**Table 1:** Descriptive data of the study population.

Variables	Males (14,148)	Females (9858)	P value
Age (years)	37.7 ± 11.7	38.0 ± 12.1	0.69
BMI (kg/m^2^)	24.1 ± 3.1	22.03 ± 3.1	< 0.001
Systolic blood pressure (mmHg)	124.7 ± 15.3	117.8 ± 16.2	< 0.001
Diastolic blood pressure (mmHg)	76.5 ± 10.8	71.3 ± 10.0	< 0.001
eGFR (mL/min/1.73 m^2^)	98.5 ± 15.4	110.1 ± 19.4	< 0.001
Total cholesterol (mmol/L)	4.46 (3.93–5.02)	4.33 (3.83–4.92)	< 0.001
Triglycerides (mmol/L)	1.27 (0.89-.187)	0.85 (0.63–1.2)	< 0.001
Total bilirubin (umol/L)	14 (11.2–17.7)	11.6 (9.4–14.6)	< 0.001
Glucose (mmol/L)	5.2 ± 0.52	5.1 ± 0.47	0.073
Albumin (g/L)	47.8 (46.2–49.4)	46.1 (43.8–47.9)	< 0.001
Uric acid (umol/L )	378.7 (148.3–434.5)	260 (150.4–301.0)	< 0.001
BUN (mmol/L)	4.6 (4.0–5.5)	4.1 (3.4–4.8)	< 0.001

*BMI* body mass index; *eGFR* estimated glomerular filtration rate.

### The relation between BUN values and age

**Figure 2:**
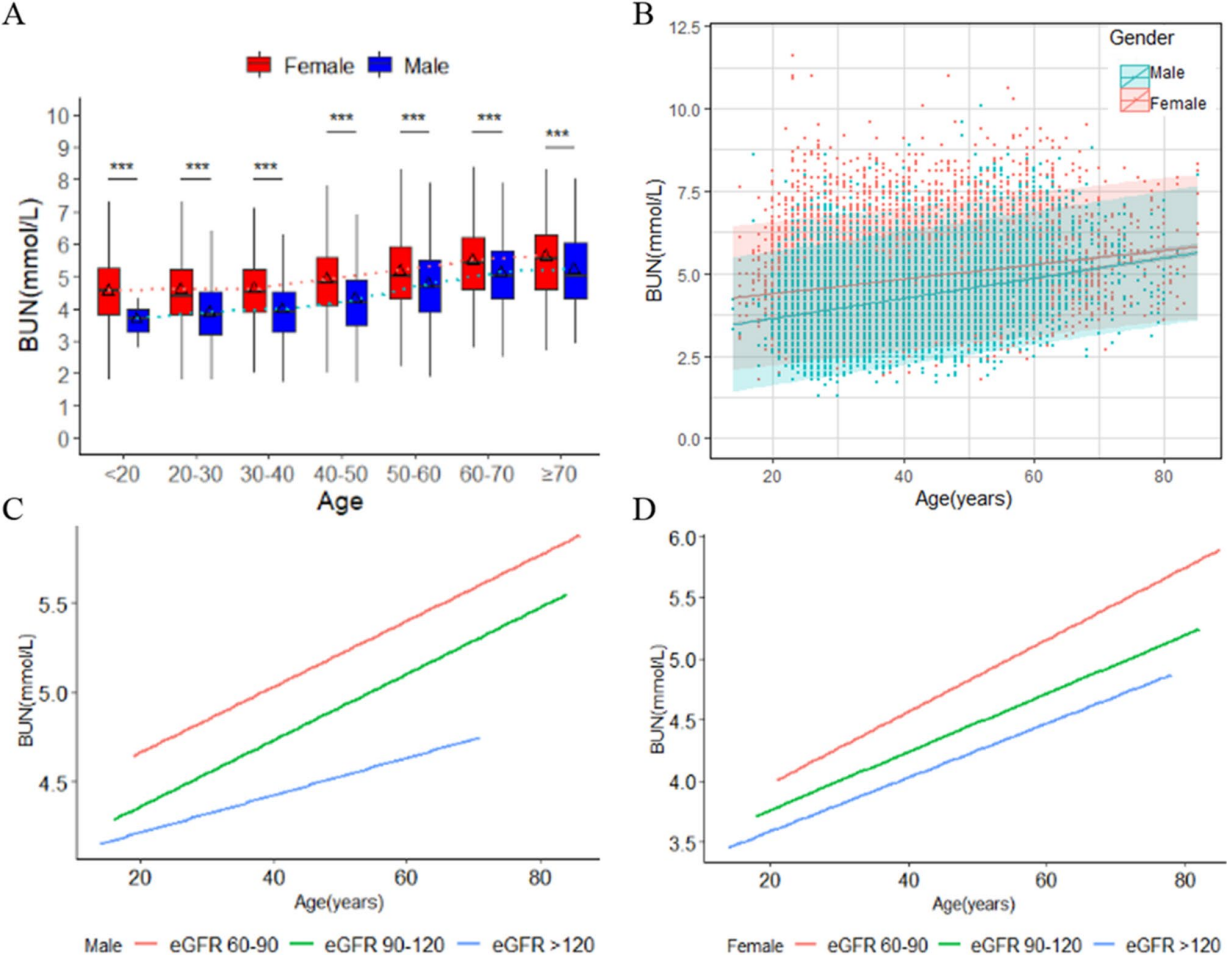
Sex-specific association between age and blood urea nitrogen (BUN). (**A**) Sex-specific boxes plots of BUN by age groups in males and females population. The black triangle in the box plots indicates the mean value. The dotted light red line represents the mean value line for each males age groups. The dotted light green line represents the mean value line for each females age groups. The definition of red boxes represent males population and blue boxes represent females population, ***P < 0.001. (**B**) Scatter plot of association between age and BUN by sex in males and females population. Solid lines express predicted BUN by age and sex with 95% prediction intervals (shaded area) for each sex. (C,D) Sex-specific associations between BUN levels and age by groups of estimated glomerular filtration rate (eGFR) in the males and females population assessed by linear regression.

As showed in Fig. 2A, the mean BUN level varied and increased steadily with age in males and females populations. In both genders, there were significant differences in BUN levels between different age groups (P < 0.0001). In brief, the estimated mean levels of BUN increased by 1.5 mmol/L from 3.7 mmol/L (95% CI 3.37 mmol/L–4.03 mmol/L) in the youngest female group to 5.2 mmol/L (95% CI 4.94 mmol/L–5.49 mmol/L) in the oldest. Similarly, ANOVA revealed up to 1.06 mmol/L higher estimated mean levels of BUN in the oldest age group in male populations, 4.56 mmol/L (4.31 mmol/L–4.81 mmol/L) compared to the youngest, 5.62 mmol/L (5.38 mmol/L–5.86 mmol/L). The rate of increase in the mean serum BUN value was 0.228 mmol/L/y, and 0.282 mmol/L/y in males and females, respectively. Linear regression analysis was carried out between age and BUN values for males and females, respectively, because there was a significant interaction between BUN values and age in both sex (P < 0.001) (Fig. 2B). Moreover, after adjusted eGFR, the relationship between BUN values and age remain unchanged in both males and females group (Fig. 2C,D). These results indicated BUN values increased stably with age in both sex.

### Correlation analysis between BUN values with other clinical variables

According to the correlation coefficient (*r*) in both sex groups, BUN values showed positive correlations with body mass index (BMI), cholesterol, and blood sugar (P < 0.0001) (Table 2). However, eGFR showed a negative correlation with the BUN reference values (P < 0.0001). Interestingly, the triglycerides, total protein, and albumin showed significantly negative correlations with BUN values in the male group, while a positive correlation in the female group. However, the relationship between BUN and eGFR did not change with additional adjustment of age and BMI, in both males and females groups. BUN values were not significantly related to blood uric acid (UA) in males groups (P = 0.23) but showed a significant positive correlation in females group (P < 0.0001).

**Table 2:** Correlation between BUN values and clinical/biochemical variables in Chinese healthy adults.

Variables	Males		Females	
	Correlation coefficient	*P* value	Correlation coefficient	*P* value
Age (year)	0.228	< 0.0001	0.337	< 0.0001
BMI (kg/m^2^ )	0.052	< 0.0001	0.122	< 0.0001
eGFR (mL/min/1.73 m^2^)	− 0.231	< 0.0001	− 0.286	< 0.0001
Cholesterol (mmol/L)	0.096	< 0.0001	0.189	< 0.0001
Triglycerides (mmol/L)	− 0.02	0.024	0.025	0.029
Blood UA (mmol/L)	− 0.011	NS	0.111	< 0.0001
Total protein (g/L)	− 0.056	< 0.0001	0.033	0.0063
Albumin (g/L)	− 0.051	< 0.0001	0.034	0.0059
Blood sugar (mmol/L)	0.090	< 0.0001	0.136	< 0.0001

*BMI* body mass index; *eGFR* estimated glomerular filtration rate; *UA* uric acid; *NS* no significance.

### Multiple linear regression analysis of the relationship between BUN values and clinical variables

Collinearity diagnostics among clinical variables were analyzed to choose the most suitable model. All the VIF values of clinical items were below 10 in both males and females, indicating that no collinearity existed in these clinical variables (Supplementary Table S1). Therefore, a linear model was used in this study. Multiple linear regression analysis confirmed that the positive associations of BUN levels and age were statistically significant after adjusting confounding factors in males (β = 0.21, 95% confidence interval, CI 0.187–0.233, *P* < 0.001), and females population (β = 0.282, 95% CI 0.255–0.309, *P* < 0.001). Thus, the increase in the rate of serum BUN values was 0.21 mmol/L and 0.282 mmol/L per 10 years, respectively. These results indicated that BUN levels of both sex groups increased steadily with age, although additionally adjusted for BMI and eGFR, albumin, and triglyceride. Conversely, a negative association between BUN levels and eGFR was observed in males (β =  − 0.013, 95% CI − 0.015 to − 0.011, *P* < 0.001) and females (β =  − 0.007, 95% CI − 0.009 to − 0.006, *P* < 0.001) population (Table 3).

**Table 3:** Multiple linear regression analysis for the association between BUN and clinical variables in males and females population.

Parameter	Male group				Female group			
	β	95% CI	SE	*P*	β	95% CI	SE	*P*
Age/10 years	0.210	0.187–0.233	0.012	< 0.001	0.282	0.255–0.309	0.014	< 0.001
BMI	0.010	0.002–0.017	0.004	< 0.001	− 0.002	0.011–0.008	0.005	0.743
eGFR	− 0.013	− 0.015 to − 0.011	0.001	< 0.001	− 0.007	− 0.009 to − 0.006	0.001	< 0.001
UA	0.000	− 0.001 to 0.000	0.000	< 0.001	0.002	0.001–0.002	0.000	< 0.001
TBil	− 0.011	− 0.014 to − 0.007	0.002	0.102	0.115	0.082–0.148	0.017	< 0.001
TP	− 0.013	− 0.020 to − 0.007	0.003	< 0.001	− 0.001	− 0.008 to 0.006	0.004	0.832
Albumin	− 0.061	− 0.081 to − 0.042	0.010	< 0.001	0.041	0.029–0.053	0.006	< 0.001
TG	0.049	0.038–0.060	0.006	< 0.001	− 0.240	− 0.284 to − 0.197	0.022	< 0.001
FBG	0.064	0.021–0.107	0.022	< 0.001	0.096	0.038–0.153	0.029	0.001

BMI: body mass index; eGFR: estimated glomerular filtration rate; SE: standard error; UA: uric acid; TBil: total bilirubin; TP: total protein; TC: triglyceride; FBG: fasting blood glucose.

### Reference values for BUN in age- and sex-specific groups

The profiles and trends of reference intervals for BUN in different sex- and age-groups are tabulated (Table 4). The BUN values in the lower 2.5th percentiles of each age male group were 1.68 mmol/L, 2.20 mmol/L, 2.90 mmol/L, 2.92 mmol/L, 3.00 mmol/L, 3.10 mmol/L, 3.40 mmol/L and 3.70 mmol/L, respectively. In the female group, the lower 2.5th percentiles were 2.20 mmol/L, 2.30 mmol/L, 2.40 mmol/L, 2.60 mmol/L, 2.80 mmol/L, 3.10 mmol/L and 3.07 mmol/L, respectively. For males aged between 20 and 29 years, the 97.5th percentile for BUN value was 7.00 mmol/L, increasing to 7.40 mmol/L in the age group 40–49 years and 8.30 mmol/L in people aged 60–69 years. Conversely, the 97.5th percentile for BUN value in the female group was 6.10 mmol/L, 6.70 mmol/L, and 7.70 mmol/L, respectively. The reference intervals for the male and female groups were significantly different, and males had higher BUN values than females in each age group.

**Table 4:** Blood urea nitrogen values (mmol/L) centiles of the age groups in males and females population.

Age (years)	Centile (90% confidence interval)				
	1st	2.5th	50th	97.5th	99th
< 20					
Male	1.60 (1.60–3.00)	1.68 (1.60–3.10)	4.75	7.07 (6.41–7.60)	7.44
Female	2.20 (2.20–2.80)	2.20 (2.20–2.80)	3.80	5.62	6.21
**20**–**29**					
Male	2.60 (2.60–2.70)	2.90 (2.80–2.90)	4.40	7.00 (6.81–7.10)	7.78 (7.50–8.10)
Female	2.00 (1.90–2.10)	2.30 (2.20–2.30)	3.80	6.10 (6.00–6.30)	6.70 (6.40–7.00)
**30**–**39**					
Male	2.61 (2.60–2.70)	2.92 (2.80–2.92)	4.50	7.00 (6.90–7.10)	7.60 (7.40–7.70)
Female	2.20 (2.10–2.20)	2.40 (2.30–2.40)	3.90	6.30 (6.20–6.40)	6.70 (6.60–6.80)
**40**–**49**					
Male	2.80 (2.70–2.90)	3.00 (3.00–3.10)	4.80	7.40 (7.30–7.50)	8.10 (7.80–8.30)
Female	2.31 (2.21–2.50)	2.60 (2.50–2.70)	4.20	6.70 (6.50–6.87)	7.19 (6.90–7.40)
**50**–**59**					
Male	2.80 (2.70–2.90)	3.10 (3.10–3.20)	5.00	7.70 (7.65–7.85)	8.34 (8.04–8.50)
Female	2.54 (2.30–2.70)	2.80 (2.70–2.90)	4.70	7.34 (7.10–7.60)	8.10 (7.70–8.20)
**60**–**69**					
Male	3.10 (2.97–3.20)	3.4 (3.20–3.50)	5.40	8.30 (8.10–8.77)	9.00 (8.72–9.15)
Female	2.80 (2.50–3.00)	3.10 (2.90–3.30)	5.00	7.70 (7.50–7.90)	8.20 (7.80–8.50)
** ≥ 70**					
Male	3.34 (2.70–3.70)	3.70 (3.60–3.83)	5.45	8.20 (7.90–15.50)	15.99 (8.20–17.20)
Female	2.90 (2.90–3.20)	3.07 (2.90–3.45)	5.00	7.34 (7.10–8.00)	7.43

The 50th centile is shown as a point estimate only, as a reference point.

## Discussion

In this study, we formulated the first age- and sex-specific reference BUN values for healthy Chinese populations. These results suggest that BUN levels are stable and significantly higher in males compared to females irrespective of age. Moreover, BUN reference values increased with age in the healthy population up to around 70–86 years for both healthy adult males and females. The relationship between BUN and age remained unchanged in both males and females groups, even though adjusted for eGFR and BMI.

The concentration of urea nitrogen in blood or serum is related to protein intake, endogenous protein catabolism, hydration state, hepatic urea synthesis, and renal urea excretion. BUN is widely used in daily clinical practice as one of the serum markers to estimate renal function and predict cardiovascular events caused by acute heart failure^12^. Matsue et al. found that BUN increased with age in males and females from the general population (4484 subjects) without cardiovascular comorbidities^12^, which was consistent with our results. Another study showed that 19 young patients had lower mean plasma urea 18 ± 8 mg/dL compared to 15 older subjects (27 ± 7 mg/dL)^13^. However, few studies have reported that normal reference levels vary with age and sex.

Although age-associated glomerular function impairment affects BUN excretion resulting in lower fractional urea excretion with age^13^, this present study excluded population with eGFR < 60 mL/min/1.73 m^2^. Moreover, the relationship between BUN and age remained unchanged in both male and female groups after being adjusted for BMI and eGFR. Thus, suggesting that the age-related increase of BUN was not affected by eGFR but related to decreasing fractional urea excretion with age. Further fundamental research is needed to elaborate on the exact mechanisms involved in the increase of plasma urea with age. The normal range of urea nitrogen in blood or serum is 5 to 20 mg/dL or 1.8 to 7.1 mmol urea per liter^14^. Thus, physicians must not misdiagnose renal dysfunction for observing plasma BUN > 8.0 mmol/L in healthy elderly.

Urea is the primary metabolite derived from dietary protein and tissue protein turnover. A significant interaction was observed between age and sex in BUN. Age-related BUN is higher in females than males, while sex-related differences for protein synthesis can partially explain sex differences in BUN levels. A study showed that females, irrespective of their muscle mass, BMI, and age, have higher fractional synthesis rates of muscle proteins and higher whole-body protein turnover. Protein metabolism does not appear to be related to the change of androgens with age^6,7^. These findings support our results of sex differences in age-related change in BUN.

BUN is a simple clinical variable and useful prognostic information for patients admitted for decompensated heart failure^15^. Fonarow et al. used the acute decompensated heart failure national registry database and identified the BUN level of 43 mg/dL as the best prognostic biomarker discriminator between hospital survivors and non-survivors^16^. The BUN level of every 10 mg/dL above a normal cutoff (17 mg/dL) increases the death risk of patients with heart failure by 21%^17^. Thus, suggesting that even mild elevation in BUN level could affect the assessment of heart failure in a population. Our study aimed to present serum BUN reference values to the Chinese population. The results showed an increase in BUN of 0.21 mmol/L per decade in males with a comparable rise in females (0.282 mmol/L per decade). Therefore, we recommend that the age- and sex-adjusted BUN reference range should be considered while diagnosing heart failure diseases in older adults to avoid over-evaluation or misevaluation of poor prognosis from slightly increased BUN values.

This study has several significant limitations. Firstly, the study was retrospective and lacked data on dietary protein intake, nutritional status, and muscle mass from males and females of the same age group. Whether these additional factors can affect BUN values were not assessed in both sex. Women's menstrual cycle, oral contraceptives and HRT can regulate female hormone levels, and a recent study has shown that hormone levels can affect BUN levels. We lack relevant data to further analyze the BUN reference interval of women at different stages of the menstrual cycle and exclude the interference of hormone levels^18^. We lack data to further analyze the reference intervals of blood urea nitrogen in women at different stages of the menstrual cycle. Secondly, this is a single-center study, and the datasets primarily consisted of subjects of Han nationality. Thus, the results do not represent the general population and may not apply to other ethnicities or regions. Recently, a study reported that the BUN reference value is significantly affected by geographical environment and location, such as latitude, altitude, annual mean temperature, annual mean relative humidity, and annual precipitation. BUN reference values of healthy Chinese adults are lower in the east and higher in the west^5^. As the elderly over 70 years rarely undergo a routine health check, their number is relatively insufficient, which could be another limitation. Thus, our results on the normal BUN reference range of a population with over 70 years old may not have enough power to evaluate this age group.

## Conclusions

In this study, we report that the reference values of serum BUN were age- and sex-related variable, and varied widely in the general population. This study is the first set of comprehensive and robust age- and sex-specific reference intervals determined for BUN in the general Chinese population. Our results clearly showed the importance of age and sex while assessing a patient's condition based on BUN values.

## Supplementary Information

Supplementary Information.
