# Author Correction: Age- and sex-specific reference intervals for blood urea nitrogen in Chinese general population

**DOI:** 10.1038/s41598-022-04908-6

**Published:** 2022-01-11

**Authors:** Qingquan Liu, Yiru Wang, Zhi Chen, Xiaolin Guo, Yongman Lv

**Affiliations:** 1 Department of Nephrology, Tongji Hospital, Tongji Medical College, Huazhong University of Science and Technology, Wuhan, 430030 People’s Republic of China; 2 Department of Geriatrics, Tongji Hospital, Tongji Medical College, Huazhong University of Science and Technology, Wuhan, 430030 People’s Republic of China; 3 Department of Urology, Tongji Hospital, Tongji Medical College, Huazhong University of Science and Technology, No.1095 Jiefang Avenue, Wuhan, 430030 Hubei People’s Republic of China; 4 Department of Health Management Centre, Tongji Hospital, Tongji Medical College, Huazhong University of Science and Technology, Wuhan, 430030 People’s Republic of China

Correction to: *Scientific Reports* 10.1038/s41598-021-89565-x, published online 12 May 2021

The original version of the Article contained an error in the Author Information section.

“These authors contributed equally: Qingquan Liu, Yiru Wang, Xiaolin Guo and Yongman Lv.”

now reads:

“These authors contributed equally: Qingquan Liu and Yiru Wang. These authors jointly supervised this work: Xiaolin Guo and Yongman Lv.”

The original Article has been corrected.

