# Supplementary material for: Age- and sex-specific reference intervals for blood urea nitrogen in Chinese general population
Source: Sci Rep. 2021 May 12;11:10058. doi: 10.1038/s41598-021-89565-x (PMC8115101; doi:10.1038/s41598-021-89565-x)
Supplement: Supplementary file 1 — Supplementary Information. [file 41598_2021_89565_MOESM1_ESM.docx]

**Age- and sex-specific reference intervals for blood urea nitrogen in Chinese general population**

Qingquan Liu^1#^, Yiru Wang ^1#^, Zhi Chen^2^, Xiaolin Guo^3*^, Yongman Lv^1, 4^*

^1^Department of Nephrology, Tongji Hospital, Tongji Medical college, Huazhong University of Science and Technology, Wuhan 430030, P.R. China.

^2^Department of Geriatrics, Tongji Hospital, Tongji Medical College, Huazhong University of Science and Technology, Wuhan 430030, P.R. China

^3^Department of Urology, Tongji Hospital, Tongji Medical College, Huazhong University of Science and Technology, Wuhan, Hubei 430030, P.R. China.

^4^Department of Health Management Centre, Tongji Hospital, Tongji Medical College, Huazhong University of Science and Technology, Wuhan 430030, P.R. China.

^#^These authors have contributed equally to this work and should be considered co-first authors.

^*^These authors have contributed equally to this work and should be considered joint co-corresponding author

Corresponding Author: Xiaolin Guo, E-mail: guoxiaolin0488@sina.com

Yongman Lv, E-mail: [lvyongman@126.com](mailto:lvyongman@126.com)

Address: No.1095 Jiefang Avenue, Wuhan, Hubei, 430030, China; Tel: +86 027- 83662848;

Supplement table 1 The VIF values of clinical variables in males and females groups.

| Variables | Males VIF | Females VIF |
| --- | --- | --- |
| Age (year) | 1.561220 | 1.849751 |
| BMI (kg/m^2^) | 1.246020 | 1.462237 |
| eGFR (mL/min/1.73 m^2^) | 1.218991 | 1.367025 |
| Uric acid ( mmol/L) | 1.268595 | 1.302320 |
| TC(mmol/l) | 1.065373 | 1.307752 |
| Total protein (g/L) | 1.315859 | 1.352003 |
| Albumin(g/L) | 1.190582 | 1.502977 |
| Triglycerides ( mmol/l) | 1.638187 | 1.384095 |
| Blood sugar (mmol/L) | 1.111776 | 1.177923 |
